# Professional Honorarium

**Published:** 1879-09

**Authors:** 


### Editorial.

### PROFESSIONAL HONORARIUM.

The customs of society in this and other countries, as to remuneration for services, press more onerously upon physicians than, perhaps any other class of professional men. As a body, the world looks upon them as humanitarians; and therefore, regards a failure to grant succor to the indigent, promptly, a criminal dereliction, meriting the condemnation of all good citizens. This is more particularly the case in rural districts, where all take cognizance of an invalid neighbor. In cities, where provision is made by the municipal authorities for the pauper population, and where the suffering of a next-door neighbor is often unknown, the demand is less urgent. Even under the most favorable circumstances, however, there is a general feeling that professional obligations require gratuitous attendance upon the poor.

Now, it is often the case that men will withhold their patronage from the inhumane doctor who will not give his services and medicine without remuneration, and at the same time feel no compunction in refusing to furnish provisions without pay. The medical man's profession is his dearly bought capital, and is as much his stock in trade as the merchant's wares, or the banker's money, and one has just the same moral obligation to divide it amongst the needy as the other. If the physician is compelled by public opinion to do this work, by all means, the burden should fall upon the public and not upon a few professional men. The majority of taxpayers would consider it a monstrous law which required the physician to be paid from the county treasury for pauper practice, and yet they are not at all moved with sympathy for the half dozen citizens who bear this expense.

The demand upon legislators for a law allowing garnishment of wages &c., by being physicians, if granted, will not secure the payment of medical bills. There is no possible way of collecting money from one that has nothing. Fee-bills, black-lists and guarnishments, all together, will not effect the object. A man whose income is hardly sufficient to feed and clothe his family, cannot be made to pay even moderate medical bills, because the means to do so are not in his possession. Humanity demands the services of a good physician for such people, but the requisition is as much upon the banker or merchant, as upon the worn-down physician, whose only means of living is in his practice of medicine.

